# Developing and translating a theory of informal caregiving in Sporadic Creutzfeldt-Jakob disease: a two-phase constructivist grounded theory study

**DOI:** 10.3389/fnins.2026.1905885

**Published:** 2026-08-12

**Authors:** Lauren Pourghaderi, Leslie F. Davidson, Brian S. Appleby, Patrick G. Corr

**Affiliations:** 1 Department of Clinical Research and Leadership, School of Medicine and Health Sciences, The George Washington University, Washington, DC, United States; 2 Department of Neurology, University Hospital’s Neurological Institute, Cleveland, OH, United States; 3 School of Medicine, Case Western Reserve University, Cleveland, OH, United States; 4 National Prion Disease Pathology Surveillance Center, Case Western Reserve University, Cleveland, OH, United States

**Keywords:** grounded theory, informal caregiving, knowledge translation, prion disease, Sporadic Creutzfeldt-Jakob disease

## Abstract

**Introduction:**

Sporadic Creutzfeldt-Jakob disease (sCJD) is distinct from related dementias in several key ways, influencing the informal caregiving experience. Caregiver burden is widely acknowledged as a public health concern, and the lack of sCJD-specific informal caregiving research leads to difficulties providing targeted resources to this population.

**Objectives:**

No foundational theory exists to describe the experience of informal caregiving in sCJD, which could inform decisions regarding study design and the provision of support resources to this population. This paper describes the methods used to develop a theory describing sCJD informal caregiving, translating this theory to relevant clinicians, identifying gaps in clinician knowledge of sCJD informal caregiving, opportunities for use in clinical practice, and potential barriers to knowledge translation and action.

**Methods:**

The theory of sCJD informal caregiving is developed using constructivist grounded theory methodology. Interviews and demographic surveys collect caregiver data. Interviews collect clinical participant data. The Knowledge-to-Action Framework guides both translation of the theory to clinical stakeholders, and clinical perspectives into future translation and action efforts.

**Discussion:**

Significant strides in pre-mortem testing and drug therapy research suggest a shift toward a prolonged period of caregiving with a known diagnosis, which may shift the roles of clinicians and informal caregivers alike. This research can support both an increased understanding of sCJD caregiving and the translation of such knowledge to appropriate stakeholders. The methodology described in this paper can be applied to understand informal caregiving in other rare diseases, including other forms of prion disease.

## 1 Introduction

Informal caregivers, those who care for a family member or friend in a non-professional capacity, play a critical role in supporting individuals with serious illnesses, but this role is associated with significant impacts on their physical, mental, and financial health (CDC, 2024; World Health Organization [WHO], 2025). A large body of research has examined caregiving for persons with Alzheimer’s disease and related dementias (ADRD) more broadly, but there is a dearth of research exploring the burden of informal caregivers in Sporadic Creutzfeldt-Jakob disease (sCJD) or other prion diseases, including consideration for how this experience of caregiving differs from other forms of dementia and neurodegenerative disease.

Sporadic Creutzfeldt-Jakob disease is a rare, fatal, rapidly progressive neurodegenerative dementia that occurs in the absence of known risk factors apart from age and codon 129 polymorphism (Connor et al., 2019; Knight, 2017; Sitammagari and Masood, 2023). sCJD is distinct from the broad categorization of ADRDs in several ways, including differences in prevalence, age of onset, rapid progression, symptom profile, diagnostic challenges including access to clinical expertise, and potential for transmission (Alzheimer’s Association, n.d.-b; CDC, 2026; Connor et al., 2019; Ford et al., 2019). sCJD does not have a consistent clinical staging framework, and not all cases follow the same presentation or progression, making it more challenging and less effective to provide generalized support and information to caregivers (Ford et al., 2019; Nemani et al., 2020; Thompson et al., 2014). The limited body of research that exists regarding sporadic and general CJD caregiving suggests that the burden of care is unique and higher than that of other dementia caregivers (Ford et al., 2019; Harrison et al., 2022; Kovacevich et al., 2025; Sideman et al., 2023; Uflacker et al., 2016). However, no study exists to understand or describe the sCJD informal caregiving experience holistically.

Despite these differences, there is currently no established theoretical framework to guide research or practice related to supporting informal caregiving in sCJD. In order to deliver effective support and resources to sCJD informal caregivers, the experience of this population must first be thoroughly understood. Further, because of the rapid neurodegenerative decline experienced by persons with sCJD, their informal caregivers often become the primary advocate, decision maker, and carer throughout the illness, and there is an opportunity for the experience and knowledge gained through this experience to inform the development of a theory used to guide future research and clinical practice. A theoretical framework contributes to a foundational understanding of sCJD informal caregiving and may guide considerations of future clinicians and researchers as they design future studies or interventions to support this population of caregivers and the patients they care for.

Generally, there has been an increased focus on translational approaches to understanding dementia caregiving and providing support to this population more broadly (Alzheimer’s Association, n.d.-b; Bolton, 2023; National Institutes of Health [NIH], 2023; Sampson et al., 2020). Research by Gitlin et al. (2015) and Sampson et al. (2020) suggests that acknowledgement of dementia subtype and staging, the etiology of rare dementias, and other contextual factors are important considerations when developing interventions to support dementia informal caregivers. The translational approach described in this paper allows for incorporation of complex contextual factors from both knowledge creators (caregivers) and knowledge users (clinicians).

## 2 Aims and objectives

This paper describes the objectives and methods used in an ongoing, two-phase study that aims to develop a theory to describe the experience of informal caregiving in sCJD, and to assess the factors that impact the translation of this theory to clinical knowledge users, including the perceived translatability and usability of this theory in clinical practice settings. While the theory itself is intentionally specific to sCJD and not generalizable, the methods used to generate the theory and assess translatability to ensure effective outcomes are adaptable to address similar problems of interest in other understudied caregiver populations, including other forms of prion disease. This paper aims to detail these methods to demonstrate how they will be used to address the gap in the theoretical foundation of understanding informal caregiving in sCJD, how a translational approach can bridge gaps between theoretical and clinical disciplines, and how similar problems in related fields might be addressed.

## 3 Methods

**FIGURE 1:**
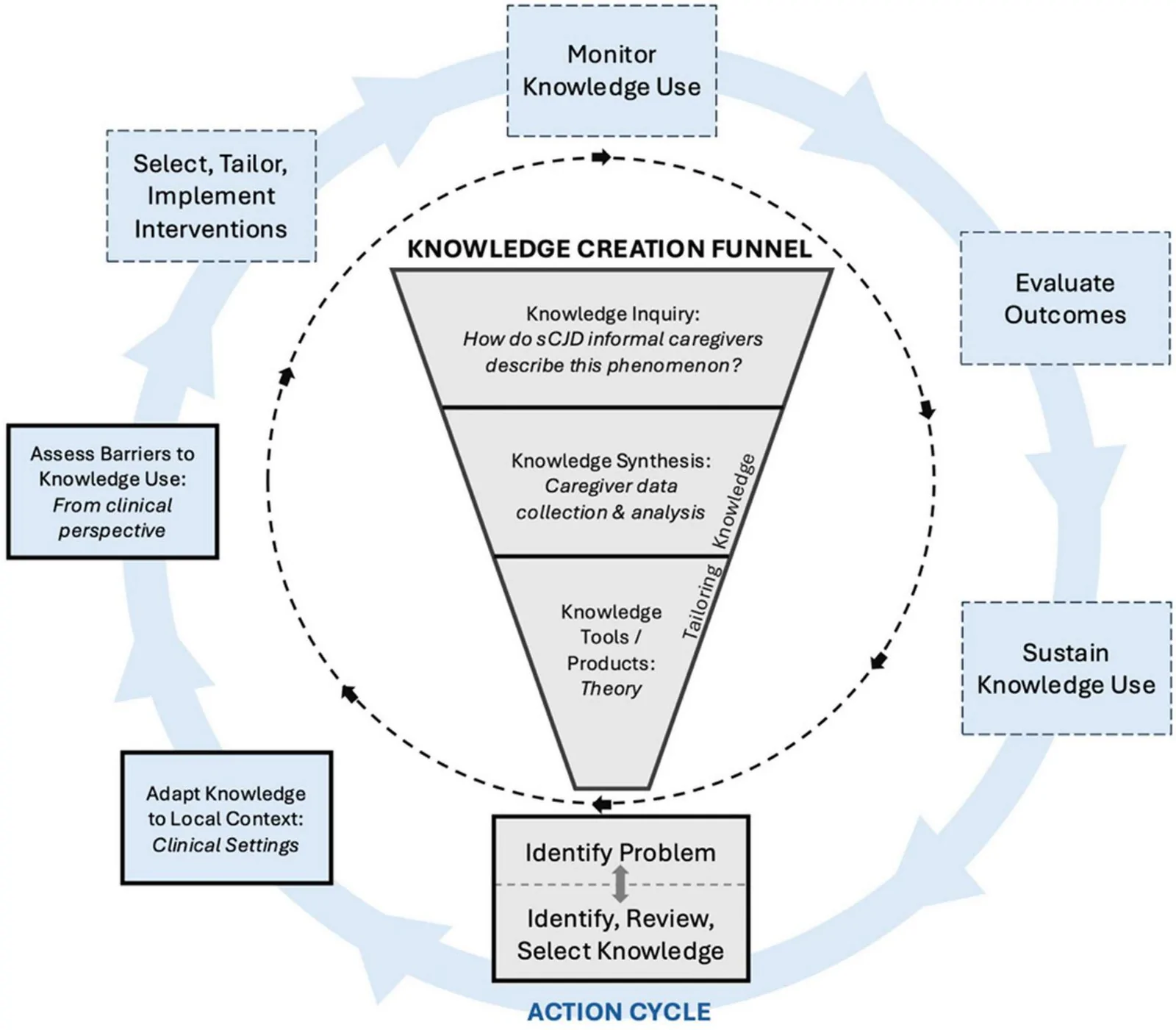
Application of the Knowledge-To-Action Framework, with solid-outlined boxes representing aspects directly addressed in this study, and dash-lined boxes representing aspects considered in study design which will be addressed directly in future research. Source: Adapted from Graham et al. (2006).

This study is guided by the Knowledge to Action framework, which guides the iterative process of knowledge creation, synthesis, translation, and use (see Figure 1; Graham et al., 2006). This framework supports the design of a study that accounts for translation and end use from inception, ensuring that the theory of informal caregiving will be effectively translated to the appropriate knowledge users, and that end-use aligns with the study’s intended goals. This study primarily focuses on the knowledge creation funnel, the junction point between the knowledge creation funnel and action cycle, and the elements of the action cycle concerned with adapting knowledge to local contexts and assessing potential barriers to knowledge use (Graham et al., 2006).

This study is structured into two phases. Phase 1 addresses the development of the theory (knowledge creation and synthesis), and is guided by the researcher question: “How do informal caregivers of persons ultimately diagnosed with probable or confirmed sCJD experience caregiving across the trajectory of illness?” Phase 2 addresses the translatability of this theory into practice and is guided by the research question: “How do clinicians involved in the care of sCJD patients perceive the usability of a theory describing the experience of sCJD informal caregivers?”

### 3.1 Phase 1–constructivist grounded theory

Phase 1 utilized a constructivist grounded theory methodology in the tradition of Charmaz (2014) in order to develop a theory of informal caregiving in sCJD. This methodology is an appropriate choice when no theory currently exists to describe a phenomenon of interest. This approach to grounded theory research acknowledges and accounts for the subjective nature of experience and interpretation on the part of both participant and researcher, emphasizing co-development of the theory between these two groups. This approach is able to account for the significant heterogeneity of experience among sCJD caregivers.

Constructivist grounded theory is characterized by an iterative process of data collection and analysis. Data analysis consists of two phases of inductive coding: initial and focused. Data is first coded line-by-line, followed by axial and theoretical coding until saturation is reached (Charmaz, 2014). The analysis of these codes results in the development of themes and identification of relationships between themes, resulting in theory development (Charmaz, 2014).

While constructivist grounded theory acknowledges the infeasibility of complete researcher separation in the practice of this methodological approach, Charmaz (2014) addresses validity concerns through the ongoing practices of memoing and researcher reflexivity (McCall and Edwards, 2021; Tufford and Newman, 2012). This study also adopted a process of member checking data and the developing theory with informal caregiver participants, and several transcripts were chosen at random to be coded by multiple researchers in order to ensure concurrence of emerging codes.

### 3.2 Phase 1–recruitment

Constructivist grounded theory stresses the importance of theoretical saturation, which cannot be guaranteed at any predetermined sample size, though qualitative researchers in this field recommend between 20 and 60 total interviews when using this methodology, in order to reach saturation (Charmaz, 2014; Creswell and Poth, 2018; Morse, 2000; Vasileiou et al., 2018). When using a constructivist grounded theory methodology, theoretical saturation is achieved once the full scope of an identified category has been addressed; any additional data reinforces the characteristics of the categories rather than adding to them (Charmaz, 2014). This is unique from the goal of reaching saturation of all possible viewpoints, types of participants, or data points. When sampling in constructivist grounded theory, Charmaz (2014) distinguishes between initial and theoretical sampling practices. While the goal of initial sampling is to recruit participants who represent the defined population of interest for a study, theoretical sampling is focused on elucidation of codes and themes that arise from the data (Charmaz, 2014). With these recommendations in mind, and with consideration for the rarity of sCJD and feasibility of study recruitment, a total of 21 participants were recruited on a rolling basis. This final participant pool was narrowed from 43 interested caregivers, 28 of whom completed the screening process, of whom six were deemed ineligible for participation (*n* = 3) or lost to follow-up (*n* = 3).

**FIGURE 2:**
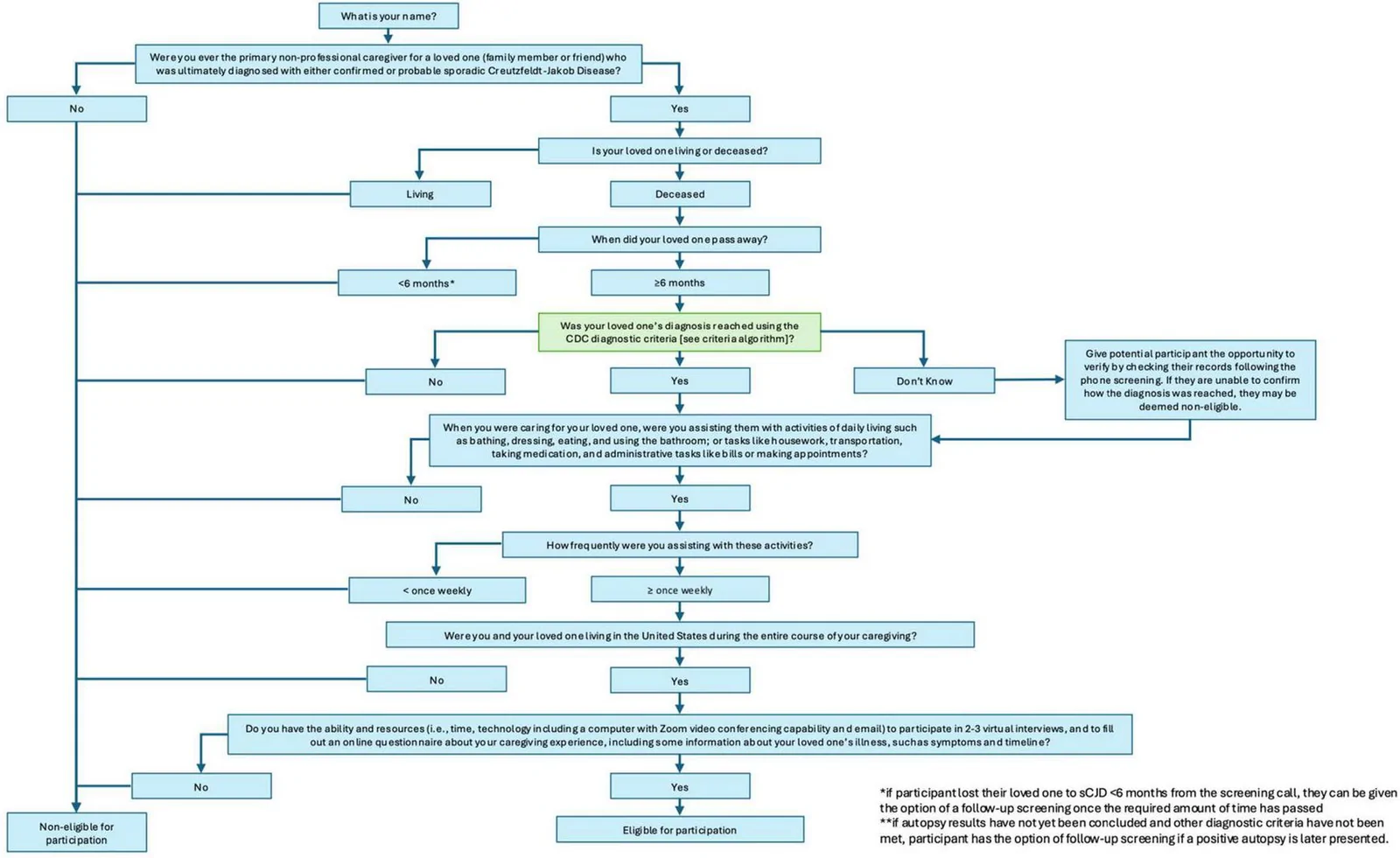
The algorithm developed to screen eligibility of potential informal caregiver participants in Phase 1 of this study.

**FIGURE 3:**
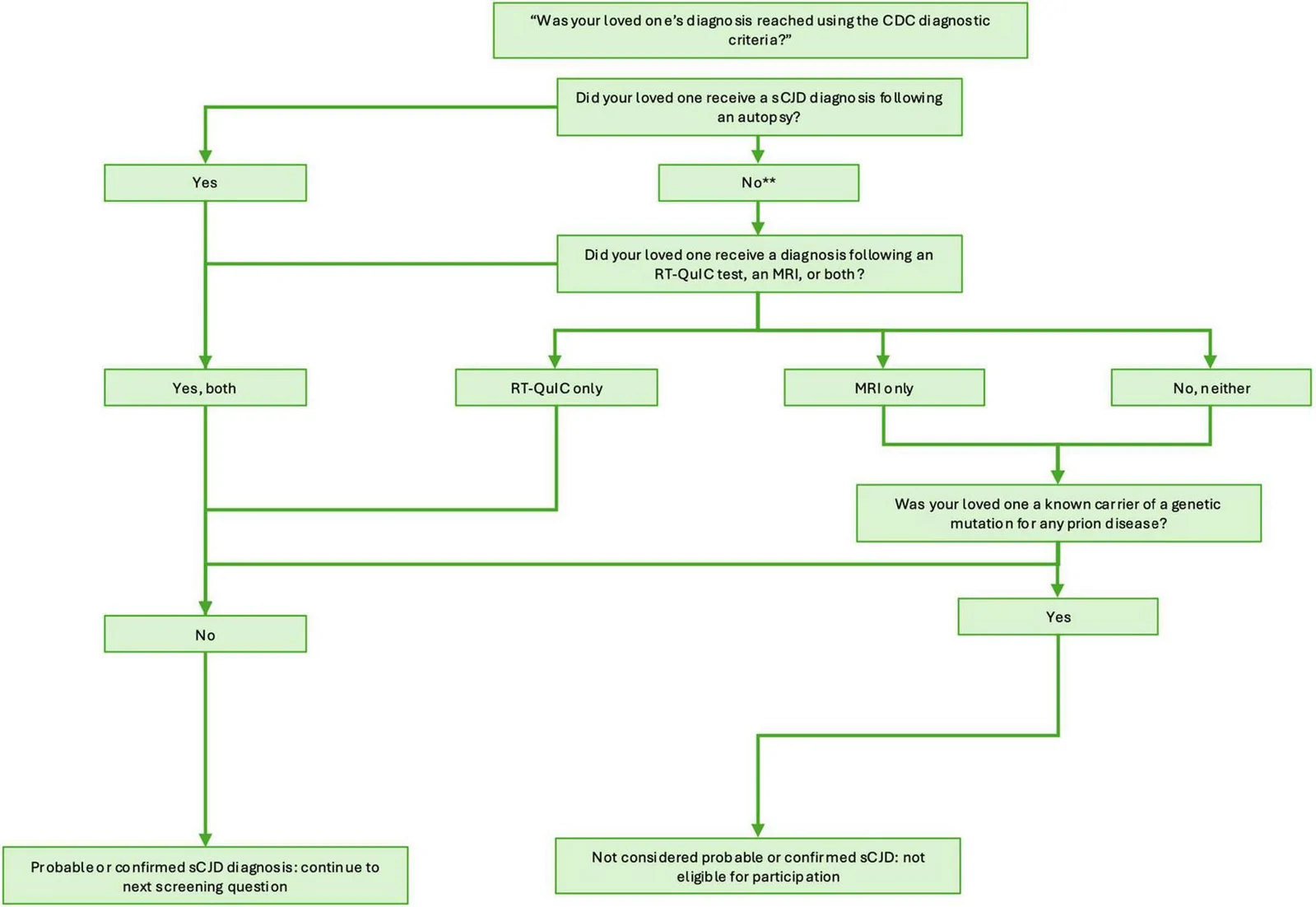
Sub-algorithm to expound upon the screening question “Was your loved one’s diagnosis reached using the CDC diagnostic criteria?” depicted in Figure 2. Participants were considered eligible if they met the criteria for probable or confirmed diagnosis of sporadic CJD.

This study recruited former caregivers of persons diagnosed with sCJD who resided along with their care recipient in the United States for the entire course of caregiving. Additionally, caregivers were eligible to participate 6 months after the death of the care recipient. There is no singular definition of informal caregiving; for the purpose of recruitment in this study, eligible participants must have provided in-person activity of daily living (ADL) and/or instrumental activities of daily living (IADL) support a minimum of once weekly throughout the care recipient’s illness (National Research Council [NRC], 2010). ADLs included abilities related to basic needs including ambulating, feeding, dressing, personal hygiene, continence, and toileting (Edemekong et al., 2025). IADLs included abilities requiring executive functioning such as transportation, managing finances and medications, shopping, cooking, managing the home, and administrative tasks (Edemekong et al., 2025). The care recipient must have ultimately received a diagnosis of either probable or definitive sCJD using the criteria specified by the CDC (2026). Eligibility was assessed with the use of a screening algorithm developed by this researcher and administered via phone call with each potential participant, available in Figures 2, 3.

Channels for recruitment included reaching out to caregiver support groups administered via academic institutions and social media groups. Ultimately, a majority of participating caregivers were contacted via a Facebook support group, and via snowball sampling.

### 3.3 Phase 1–data collection and analysis

Following the eligibility screening and consenting process, data collection consisted of two or three interviews with each participant; (1) a semi-structured virtual interview lasting 60–90 min; (2) a follow-up virtual interview held 1–4 months following the initial interview to provide both researcher and participant the opportunity to ask follow-up questions, clarify details, share additional information, and conduct preliminary member checking; (3) an optional member-checking interview where a preliminary version of the developing theory was shared with caregiver participants, with participant feedback informing the final draft.

Caregivers completed a demographic survey following the first interview which collected demographic data on both the caregiver and care recipient. Caregiver data included age, geographic region, gender and racial/ethnic identities. Care recipient data included age and year of death, diagnostic information (testing methods, identified subtype and polymorphism), timelines from first symptom to diagnosis and diagnosis to death, data regarding life-prolonging measures, and locations of care. Information about the relationship between caregiver and care recipient was collected during interviews. The purpose of the demographic survey was to provide context to the theoretical findings in Phase 1 of this study, as the product of this phase (the theory) was a representation of significant aspects of the informal caregiving experience and their dynamics, without necessarily describing or relating specific demographics.

Additional forms of data were documents provided voluntarily by some participants, including excerpts from journal entries taken during the caregiving period, and in once case the forward to a book in tribute to the care recipient and caregiver’s experience. At times, photographs were shared or described during the virtual interviews but were not shared with the researcher outside the context of the interview. The descriptions were transcribed and included in data analysis.

Data analysis was ongoing and contributed to adaptations and improvements in the way future data was collected. For example, the semi-structured interview protocol was adapted over time to account for discoveries made in earlier interviews about which questions were relevant, or things that were not addressed.

All interviews following the phone screening took place via Zoom videoconferencing software, and audio and visually recorded then transcribed (Zoom Video Communications Inc, 2026). Visual recordings were reviewed for non-verbal forms of communication such as descriptive gestures and emotional shifts, which were included in the transcription.

Data analysis was conducted using Atlas.ti for Mac qualitative software (version 26.0.1) (Atlas.ti Mac, 2026). From over 500 codes identified in the initial and follow-up transcripts, 16 categories of significance emerged, and further analysis of the data led to the identification of relationships between categories, resulting in a theory. This theory was shared with member-checking participants and refined based on their feedback.

This approach grounds the development of theory in the experiences of the participant themselves, rather than drawing from existing research or prior researcher expertise or assumptions. Thus, caregiver data informed both the factors and relationships represented in the final theory, and the identification of key knowledge user participants for the second phase of this study, described below.

### 3.4 Phase 2–pilot study assessing translatability

The second phase consisted of a pilot study assessing the translatability of the developed theory from research into practice. The purpose of this pilot was to preliminarily assess the factors that may affect the translation of this theory to the appropriate clinical knowledge users (i.e., how, when, and why it might be accessed) as well as the perceived usability of this theory in clinical practice (i.e., how, when, and why action may or may not be taken based on the information). This pilot protocol consisted of semi-structured virtual interviews with key knowledge users from each of the identified clinical specialties.

### 3.5 Phase 2–recruitment

**FIGURE 4:**
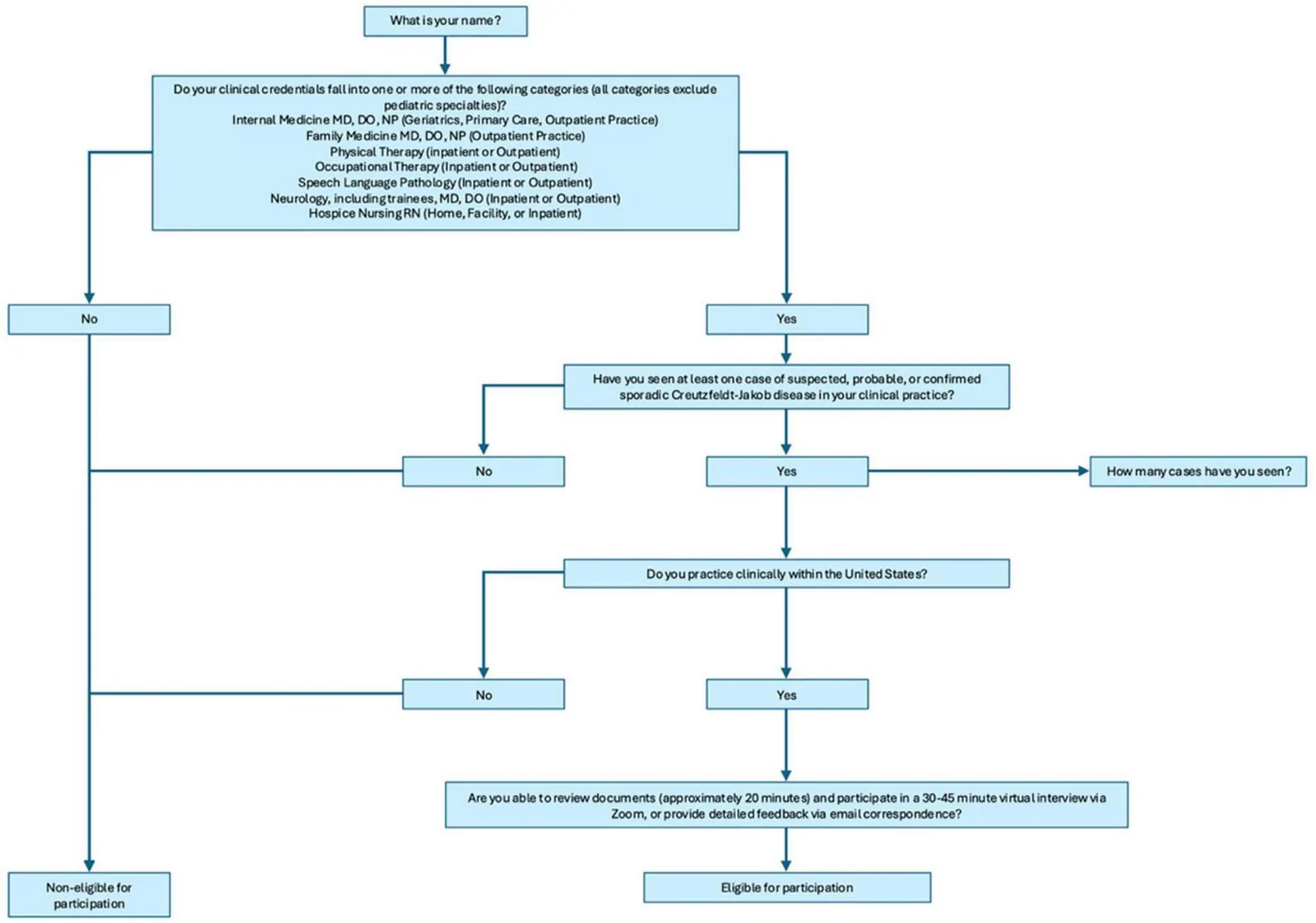
The algorithm developed to screen eligibility of potential clinical knowledge user participants in Phase 2 of this study.

The identification of key clinical knowledge users emerged from the caregiver data collected in Phase 1. Because this theory is grounded in the experiences of the caregivers, it was important to base recruitment on who played a significant role in the caregiving experience, rather than drawing conclusions based on clinicians who specialize in prion diseases. Clinical knowledge users were identified based on the frequency with which they were mentioned and utilized; the contextual significance of interactions with caregivers (e.g., first medical point of contact, provider of crucial information, trusted or established relationship); and whether they were directly identified as significant by the caregiver. These clinical knowledge users fell into four categories: primary care/family medicine (MDs, DOs, NPs in outpatient primary care practice); rehab services (physical, occupational, and speech therapy), neurology (MDs and DOs in both inpatient and outpatient settings); and hospice nursing (RN) (home, facility, and inpatient settings). In addition to the necessary board certifications and/or credentials, participants for this phase must have seen at least one case of suspected, probable, or confirmed sCJD in clinical practice, and must practice in the United States. The full screening algorithm can be found in Figure 4.

Recruitment of clinical knowledge users included purposeful and snowball sampling. Recruitment information was distributed via email, both directly and via newsletters, professional discussion forums and Facebook groups, and LISTSERV groups. Presentations were made to committees that were likely to attract the population of interest. A referral system was coordinated with the National Prion Disease Pathology Surveillance Center (NPDPSC) in which relevant clinicians were made aware of the study if they called in with questions. A system of referrals within the researchers’ own professional networks was also utilized.

Recruitment for this phase included an eligibility screening conducted via phone or email, followed by the same process of informed consent conducted in Phase 1. Because this is a pilot study, the recruitment target was between two and four clinicians per specialty area (primary care, neurology, hospice, rehab services). However, after exhausting many avenues for recruitment over several months, time and feasibility constraints resulted in a final recruitment of one clinician per category. Ultimately, this pilot study included one primary care Nurse Practitioner, one neurologist, one speech language pathologist, and one hospice nurse. Future studies seeking to explore translation of this theory into practice should look to expand this target in order to account for individual, regional, and system differences that may affect translation efforts, with an acknowledgement of the difficulties that may be faced in reaching clinicians who fit the study criteria.

### 3.6 Phase 2–data collection and analysis

Following the eligibility screening and consenting process, data collection will consist of one virtual, semi-structured interview conducted virtually via Zoom. At least 1 week prior to the interview, each clinician will receive a copy of the sCJD informal caregiving theory via email to review. The interview will address the clinician’s overall thoughts regarding the theory, their perspectives on usability, barriers and facilitators to receiving the information, opportunities for taking action in clinical practice to better support informal caregivers, and perceived barriers to action based on their experience of the clinical practice environment.

All interviews will be recorded and transcribed via Zoom (Zoom Video Communications Inc, 2026). Clinicians will also be offered the opportunity to correspond via email if a Zoom interview is not feasible. Zoom and email interview transcripts will be analyzed based of the categories of translatability and usability described above. Findings will inform future actions taken to translate this theory, as well as future research into the impact of local clinical contexts on the translation of sCJD informal caregiving research into practice.

## 4 Trustworthiness and rigor

To ensure trustworthiness and rigor in this study, a random selection of transcripts were coded by either two or three members of the research team. Researcher reflexivity and memoing, two practices characteristic of grounded theory research, were ongoing and required the primary researcher to acknowledge and separate personal biases, allowing findings to emerge directly from the data (Charmaz, 2014). Member checking with caregiver participants ensured that analysis and theory development were aligned with their experiences, allowing the researcher to validate what was coded from earlier interviews, and to receive feedback on the emerging theory and coinciding model. This contributed to the iterative process of theory development and readiness for translation to clinical participants (Charmaz, 2014; Creswell and Poth, 2018).

Initial member checking occurred as part of the follow-up interviews, where each participant was asked for clarification or validation of certain things they discussed in their initial interviews, as well as their thoughts on emerging codes, categories, and themes early in the process of theory development. In the optional, dedicated member checking interviews, participants were asked for their overall impressions and thoughts on the theory, which was shared with them in advance of the interview, as well as their perspectives on which aspects of the theory aligned most and least with their own experience, whether they felt that any components of their experience were missing or misrepresented, and what they believed the intended impression of the theory should be on clinical knowledge users. By conducting member checking at two points throughout the process of theory development, the researchers and caregiver participants had multiple opportunities to ensure alignment of the caregivers’ experiences and perspectives with the emerging theory, feedback with was incorporated both into the analysis itself, and into the representation and delivery of the information to clinical participants during Phase 2 (Busetto et al., 2020).

## 5 Ethical considerations

The sensitivity of the subject matter addressed in this study was acknowledged throughout for both informal caregiver and clinical participants. Members of either participant group may experience emotional distress when reflecting on their experiences providing care. This was addressed in the informed consent document, which included contact information for the CJD Foundation, which is the preeminent organization for prion disease caregiver support in the United States.

Due to the nature of this qualitative research, complete anonymity was not possible, which introduced risks to privacy and confidentiality. This was addressed through deidentification of participant data in both interview transcripts and demographic surveys which were designed to avoid the necessity of data collection that would identify individuals and included a coded link process to avoid the necessitation of providing names or contact information as part of the survey. Data was securely stored on a password-protected cloud-based content management software.

A process of informed consent was conducted with each participant deemed eligible following the screening call. To ensure comprehension and ongoing consent, the primary researcher began each participant’s initial virtual interview with a verbal review of the consent details and participant rights, and participants were given the opportunity to ask clarifying questions (National Commission for the Protection of Human Subjects of Biomedical and Behavioral Research [NCPHS], 1979). Institutional Review Board approval was obtained through the George Washington University [IRB #NCR245728].

## 6 Limitations

The methods used for recruitment retrospective nature of the study introduced the potential for self-selection and recall bias into both phases of the study (Creswell and Poth, 2018; Portney, 2020). One contributor to recall bias in this study was the wide range of timeframes during which participants in both phases, particularly informal caregivers, cared for a person with sCJD. In this study, caregiving took place at a range of 6 months to 24 years from the time of data collection. This range could have affected how participants recalled and described experiences in this study. While Phase 1 recruitment included many avenues, the most fruitful was via Facebook, contributing to self-selection bias as participants who engage with caregiving groups on social media may be more likely to share their experience, and may have more easily accessed recruitment information for this study. The choice of a constructivist grounded theory methodology, while providing a structured approach to qualitative data collection and analysis, introduces the potential for researcher bias, which was addressed through the practices described above in the discussion on validity. Generalizability of the findings from this research to other rare diseases, including other forms of prion disease, is limited and not an intended outcome. Given the size of the participant sample and heterogeneity of the illness, findings may not be generalizable to all forms of sCJD. The sample of 21 informal caregiver participants may have limited theoretical saturation during the grounded theory analysis.

## 7 Discussion

This study describes the use of a methodology that has never been applied in the field of prion disease research and has the potential to support positive change in this field of study and clinical practice. The experiences comprising the sCJD informal caregiving phenomenon are complex and diverse, due in part to the unique nature of sCJD as a rapidly progressive disease with heterogeneous presentation. The development of a single, unifying theory that can encompass this diversity of experiences provides a foundation to inform future research and clinical decision making for clinicians and researchers who engage with individuals diagnosed with sCJD and their informal caregivers. The application of this theory can promote effective communication, resource delivery, and support. The burden faced by sCJD caregivers is significant, and the research that exists to better understand the specific needs and experience of this population, including where this experience diverges and overlaps with other neurodegenerative or rapidly progressive diseases, is sparse. While a comprehensive analysis of how informal caregiving in sCJD is similar to or different from other forms of prion disease or other neurodegenerative diseases is outside the scope of this study, the theoretical foundation resulting from this research contributes to a more specific and comprehensive understanding of this population, which may influence future actions by clinicians and researchers in this space. The translational aspect of this study considers the usability and applicability of this work from inception and is the first step in assessing how this type of work may impact clinicians. Significant strides in pre-mortem testing and drug therapy research suggest a shift toward a prolonged period of caregiving with a known diagnosis, and potential long-term involvement by a multidisciplinary care team (Bizzi et al., 2020; Ford et al., 2019; Harrison et al., 2022; Raymond et al., 2019; Sideman et al., 2023). As such shifts occur, the roles of clinicians and their relationships to sCJD patients and informal caregivers will likely change. The product of this research can help clinicians to make informed decisions to best support and engage with this population.

Beyond the development of a theory, this study is guided by a translational framework, which accounts for the knowledge user and end-use from the initial planning phase. Considering these variables from inception promotes effective translation from research to practice (Graham et al., 2006). Research by Gitlin et al. (2015) and Sampson et al. (2020) suggests that acknowledgement of dementia subtype and staging, the etiology of rare dementias, and other contextual factors are important considerations when developing interventions to support dementia informal caregivers. This study contributes to the growing use of translational approaches in dementia caregiver research, and to the emerging field of CJD-specific caregiver research.

## Data Availability

The original contributions presented in this study are included in this article/supplementary material, further inquiries can be directed to the corresponding author.
